# Xanthohumol Prevents Atherosclerosis by Reducing Arterial Cholesterol Content via CETP and Apolipoprotein E in CETP-Transgenic Mice

**DOI:** 10.1371/journal.pone.0049415

**Published:** 2012-11-16

**Authors:** Hiroshi Hirata, Yimin, Shuichi Segawa, Moeko Ozaki, Naoyuki Kobayashi, Tatsuro Shigyo, Hitoshi Chiba

**Affiliations:** 1 Frontier Laboratories of Value Creation, Sapporo Breweries Ltd., Yaizu, Shizuoka, Japan; 2 Department of Advanced Medicine, Graduate School of Medicine, Hokkaido University, Kita-ku, Sapporo, Japan; 3 Faculty of Health Science, Hokkaido University School of Medicine, Kita-ku, Sapporo, Japan; Harvard Medical School, United States of America

## Abstract

**Background:**

Xanthohumol is expected to be a potent anti-atherosclerotic agent due to its inhibition of cholesteryl ester transfer protein (CETP). In this study, we hypothesized that xanthohumol prevents atherosclerosis *in vivo* and used CETP-transgenic mice (CETP-Tg mice) to evaluate xanthohumol as a functional agent.

**Methodology/Principal Findings:**

Two strains of mice, CETP-Tg and C57BL/6N (wild-type), were fed a high cholesterol diet with or without 0.05% (w/w) xanthohumol ad libitum for 18 weeks. In CETP-Tg mice, xanthohumol significantly decreased accumulated cholesterol in the aortic arch and increased HDL cholesterol (HDL-C) when compared to the control group (without xanthohumol). Xanthohumol had no significant effect in wild-type mice. CETP activity was significantly decreased after xanthohumol addition in CETP-Tg mice compared with the control group and it inversely correlated with HDL-C (%) (*P*<0.05). Furthermore, apolipoprotein E (apoE) was enriched in serum and the HDL-fraction in CETP-Tg mice after xanthohumol addition, suggesting that xanthohumol ameliorates reverse cholesterol transport *via* apoE-rich HDL resulting from CETP inhibition.

**Conclusions:**

Our results suggest xanthohumol prevents cholesterol accumulation in atherogenic regions by HDL-C metabolism *via* CETP inhibition leading to apoE enhancement.

## Introduction

Atherosclerosis is a complex multifactorial disease and hypercholesterolemia is a well-established risk factor for the incidence of atherosclerosis and its pathological complications. The Framingham study demonstrated that lowering cholesterol levels is a direct therapy for treatment of atherosclerosis [1]. However, it has been reported that lowering cholesterol levels is not sufficient for its treatment, because the residual risk of atherosclerosis remained unchanged in spite of statin therapy [2]. For this reason, HDL cholesterol (HDL-C) has become the recent focus as a therapeutic target [3]. It is well known that HDL plays an important role in reverse cholesterol transport (RCT) and has anti-oxidative and anti-inflammatory properties [4], [5]. Hence, a great deal of interest has been paid to the development of new therapies to increase HDL to reduce the risk of coronary heart disease. Cholesteryl ester transfer protein (CETP) catalyzes the transfer of cholesteryl esters (CE) from HDL to apolipoprotein B-containing lipoproteins (*e.g*. LDL and VLDL) [6]. Patients who are constitutively deficient in CETP have high HDL-C [7], [8] and apolipoprotein E (apoE) [9], which results in substantially increased cholesterol efflux activities [10]. Thus, CETP is involved in cholesterol efflux in RCT, and plays a crucial role in regulating HDL-C levels. These effects were also reported to be induced by a CETP inhibitor as well as congenital CETP deficiency [11], [12]. This suggests that inhibitors of CETP may act as anti-atherogenic agents [13]–[15]. CETP inhibition is expected to result in the elevation of HDL-cholesterol levels and is a promising anti-atherogenic strategy.

Another important factors associated with RCT, other than CETP, are scavenger receptor class B type I (SR-B1) and LDL-receptor (LDL-R). SR-B1 is a HDL receptor that mediates the selective uptake of HDL-C. Since rodents such as mice and rats are naturally deficient in CETP, SR-BI significantly contributes to HDL-C metabolism and atherogenesis in rodents. To address drug and foodstuff affecting CETP, CETP-transgenic mice (CETP-Tg mice) are suitable experimental model. LDL-R is a key receptor for maintaining cholesterol homeostasis by control of cholesterol uptake in mammals. Lower levels of cholesterol in the liver cells lead to an increase in the number of LDL-R molecules expressed on the cell surface, improving plasma clearance of LDL [16]. Apolipoproteins serve as templates for the assembly of lipoprotein particles, maintain their structure and direct their metabolism through binding to membrane receptors and regulation of enzyme activity. Apolipoprotein A (apoA) is the major protein component of HDL and critical for RCT [17].

Xanthohumol is a prenylated chalcone derived from natural products and reported to have many physiological properties, such as the prevention of nonalcoholic steatohepatitis [18], diacylglycerol acyltransferase inhibition [19] and anti-oxidant potential [20]. In our previous studies, we screened for CETP inhibitors from natural products and identified xanthohumol as an active compound [21]. We then hypothesized that xanthohumol’s anti-atherosclerotic property was due to inhibition of the CETP reaction. In this study, we investigated the inhibitory potency of xanthohumol against endogenous CETP activity, arterial cholesterol content and expression levels lipoprotein receptors and apolipoproteins in CETP-transgenic mice, since rodents have a congenital defect in CETP.

**Figure 1:**
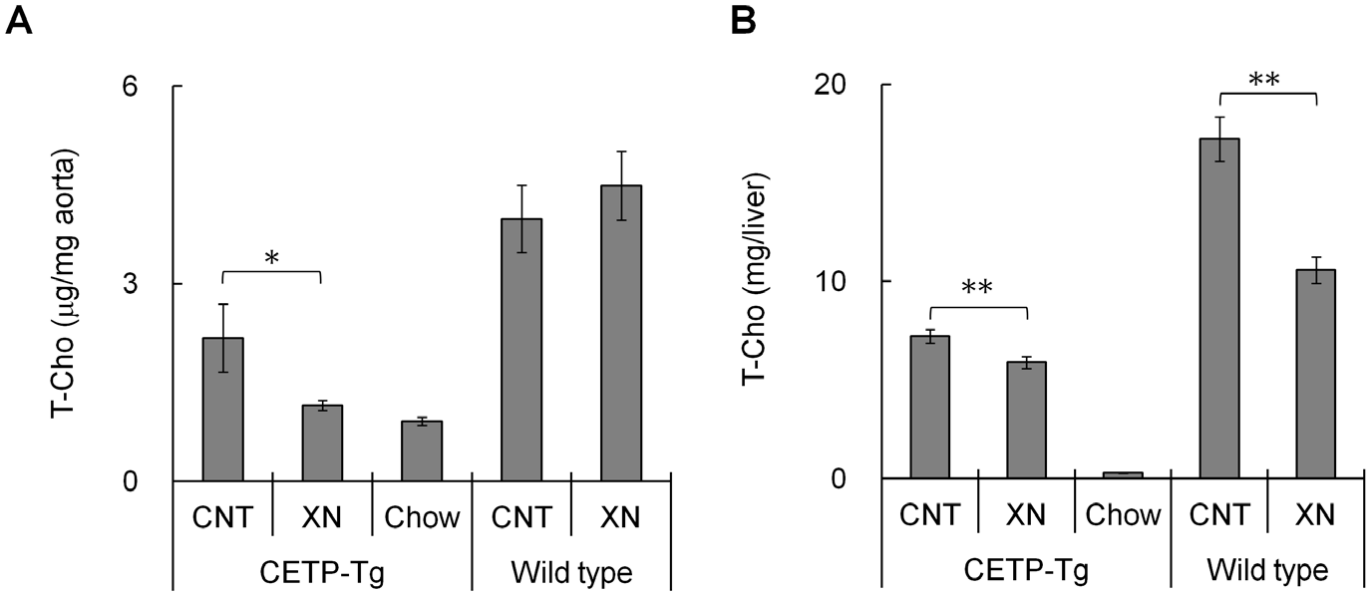
Changes in cholesterol accumulation over 18 weeks. Data are presented as cholesterol amount in the aortic arch (A) and liver (B). (N = 15; CETP-Tg mice control, N = 18; CETP-Tg mice xanthohumol, N = 12; CETP-Tg mice Chow, N = 10; wild-type mice control, N = 7; wild-type mice xanthohumol) Means±SEM. *P<0.05, **P<0.01.

## Methods

### Animals and Diets

All experiments were approved by the Hokkaido University School of Medicine Animal Care and Use Committee and were in compliance with the Guide for the Care and Use of Laboratory Animals. Female C57BL/6J and male B6.CBA-Tg (CETP) 5203Tall/J mice were purchased from SLC (Hamamatsu, Shizuoka, Japan) and Jackson Laboratory (Bar Harbor, ME, USA), respectively, for use as the parent generation (F0). F0 females were inbred with F0 males to produce first generation (F1) mice. CETP mRNA expression in 4–5 week-old F1 male mice were checked by PCR, and the CETP^+/−^ or CETP^+/+^males were used in experiments. All the mice were maintained in a temperature-controlled room with a 12-hour light/dark diurnal cycle and had free access to food during the experiment period.

CETP-Tg and C57BL/6N (wild-type) mice were subdivided into 3 and 2 groups, respectively. Throughout the experimental period, CETP-Tg and wild-type mice received an atherogenic diet containing 1% (w/w) cholesterol (high cholesterol diet, HCD). The detailed composition of the HCD is described in Table S1. In one group, HCD feeding was continued for 18 weeks (control, CNT group). The xanthohumol-fed group (xanthohumol, XN group) received the same diet as the HCD group but supplemented with 0.05% (w/w) xanthohumol powder (85% purity, Hop Steiner, Germany). Similarly, wild-type mice were fed the same diets. Chow diet (MF diet) was purchased from Oriental yeast, Ltd. and supplied to CETP-Tg mice (Chow group). The dose ratio was determined and it was found that there was no significant difference in the food intake between the with or without xanthohumol diet groups.

### Lipid and Apolipoprotein Analysis of the Plasma and HDL Fraction

Total serum cholesterol (T-Cho), HDL-C and triglyceride (TG) levels were measured every 6 weeks, using test-Wako kits (Wako Pure Chemicals). To prepare the HDL fraction, serums were mixed with an equal volume of 13% polyethylene glycol 6000 (PEG, Wako Pure Chemicals) before centrifugation (8000 *g*, 10 min, room temperature) [9]. We confirmed that the supernatants contained HDL and apoE-rich HDL by lipoprotein electrophoresis (Figure S1). Lipoprotein electrophoresis for sera and HDL-fractions were performed using agarose gel (Helena Laboratories, Texas, USA) with barbital buffer. Cholesterol and cholesterol ester were enzymatically stained.

After 18 weeks, free cholesterol and phospholipid (PL) were analyzed using test-wako kits. (Wako Pure Chemicals). ApoE concentration was measured as follows: albumin and IgG were removed from serum or supernatant prepared from 13% PEG precipitation using ProteoExtract Albumin/IgG Removal Kit (Merck). The protein concentration of the eluates was measured using the Lowry method (Bio-Rad, Ltd.) and bovine serum albumin (BSA, Bio-Rad, Ltd.) as a protein standard. Samples were then applied to SDS-PAGE/western blotting, as described below.

**Figure 2:**
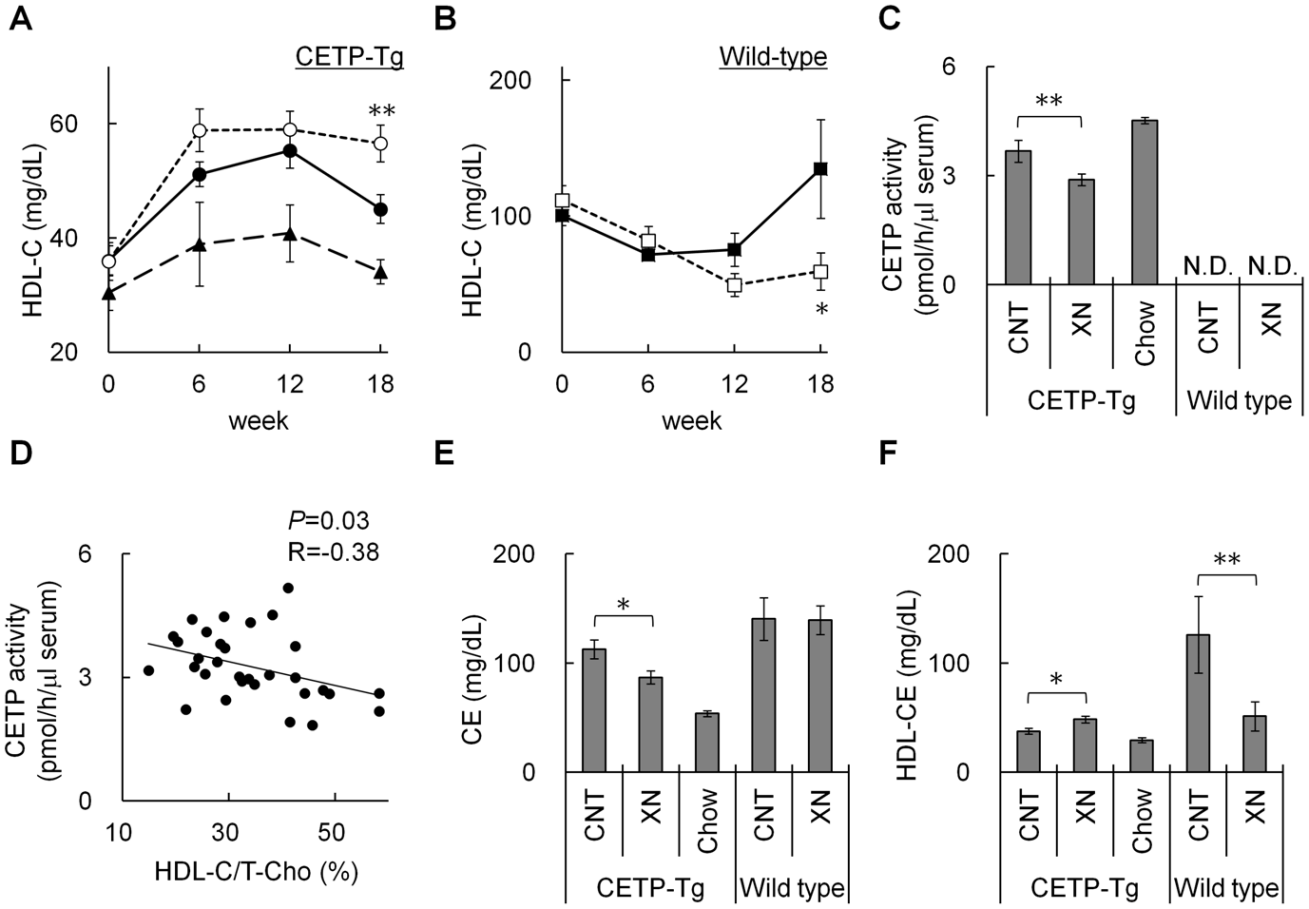
Effect of xanthohumol on serum cholesterol and CETP activity. Serum HDL-C concentration in the control group (closed circle), xanthohumol group (opened circle) and Chow group (closed triangle) of CETP-Tg mice (A), and in the control group (closed square) and xanthohumol group (opened square) of wild-type mice (B) over time. (C) Serum CETP activity after 18 weeks of treatment. (D) Correlation of serum CETP activity and HDL-C/T-Cho (%) in CETP-Tg mice fed HCD after 18 weeks. CE content of serum (E) and HDL-fraction (F) after 18 weeks. (N = 15; CETP-Tg mice control, N = 16; CETP-Tg mice xanthohumol, N = 10; CETP-Tg mice Chow, N = 3; wild-type mice control, N = 8; wild-type mice xanthohumol) Means±SEM. *P<0.05, **P<0.01.

**Figure 3:**
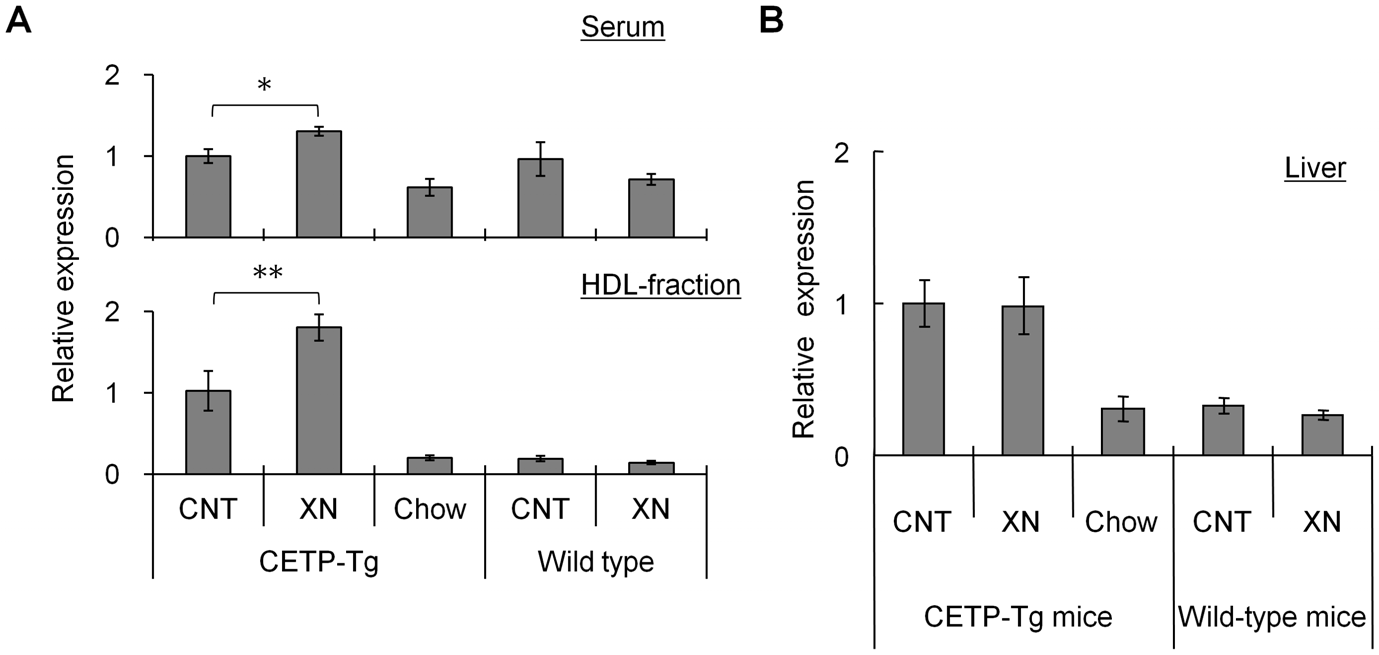
Xanthohumol increased apoE protein expression in CETP-Tg mice after 18 weeks. (A) Serum (upper) and HDL-fraction (lower), and (B) liver tissue. Data are represented as relative expression: the CETP-Tg mice control group was set at 1.0. (N = 8; CETP-Tg mice control, N = 8; CETP-Tg mice xanthohumol, N = 3; CETP-Tg mice Chow, N = 6; wild-type mice control, N = 7; wild-type mice xanthohumol) Means±SEM. *P<0.05, **P<0.01.

### CETP Activity Assay

CETP activity was measured using a CETP activity assay Kit (Bio-Vision Inc.) with minor modification [21]. CETP activity was determined by measuring the lipid transfer in a reaction mixture containing 75 µl of water, pre-incubated at 37°C, 5 µl of serum and 20 µl of master mix solution, composed of 10 µl of 10×buffer, 5 µl of donor and 5 µl of acceptor molecules. It was mixed and dispensed at 20 µl each per well (384 well fluorescence plate, Greiner Bio-One) and pre-incubated at 37°C. The transfer reaction was immediately monitored for 15 min at one minute intervals and detected using a fluorescence plate reader (Corona Electric, Ltd) (excitation at 465 nm, emission at 530 nm).

### Cholesterol Content in the Aortic Arch and Liver

After 18 weeks of treatment, mice were euthanized to collect the liver and aortic arch. The tissues were washed with PBS and weighed. Liver pieces (0.50 g) were homogenized in 5 mL methanol before 10 mL chloroform was added and homogenized [22]. The homogenates were left for at least an hour at room temperature. After filtration, the filtrates were made up to 20 mL with chloroform/methanol (2∶1, v/v), and then a solvent containing an equal volume of 5% cholic acid (Wako Pure Chemicals, Japan) dissolved in methanol was added before samples were dried at 60°C for enzymatic analysis of T-Cho, TG and phospholipid. Aortic arches were homogenized in 1 mL chloroform/methanol (2∶1, v/v) and the above procedure was then performed before enzymatic determination of T-Cho.

**Figure 4:**
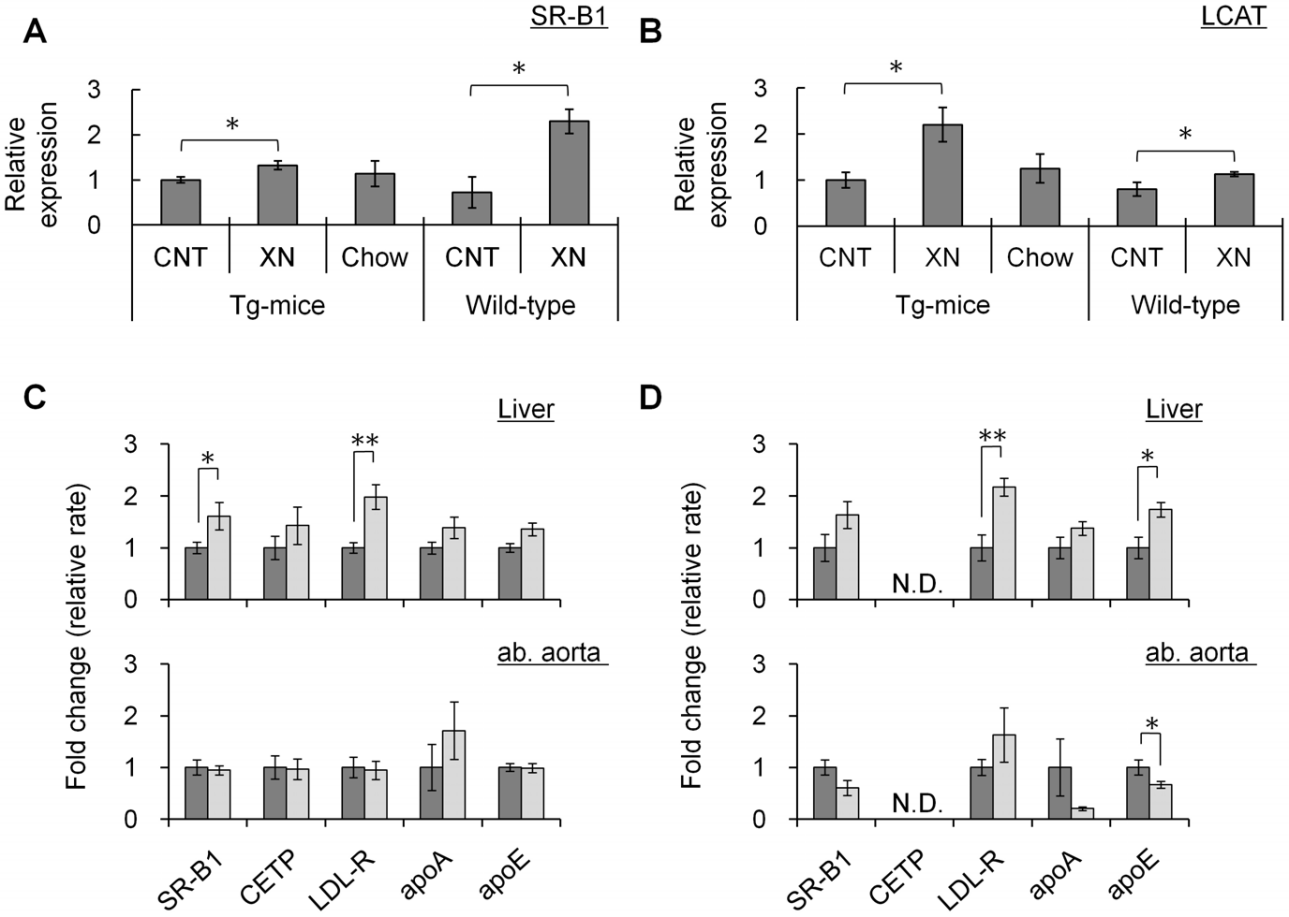
Expression analyses in mice liver and ab. aorta. (A) SR-B1 and (B) LCAT protein expression in liver. Data was standardized for β-actin expression. (N = 15; CETP-Tg mice control, N = 18; CETP-Tg mice xanthohumol, N = 11; CETP-Tg mice Chow, N = 6; wild-type mice control, N = 8; wild-type mice xanthohumol) (C, D) Transcript analyses of liver (upper) and ab. aorta (lower) in CETP-Tg mice (C) and wild-type mice (D). (N = 12; CETP-Tg mice control, N = 13; CETP-Tg mice xanthohumol, N = 9 to 10; CETP-Tg mice Chow, N = 5; wild-type mice control, N = 7 to 8; wild-type mice xanthohumol) All data were standardized for GAPDH expression. Expression levels of control group (without xanthohumol) were set at 1.0. Means±SEM. *P<0.05, **P<0.01.

### Western Blotting Analyses

The excised livers were transferred into cold RIPA buffer (Wako Pure Chemicals) on ice and then homogenized and centrifuged (20,000 *g*, 5 min, 4°C). The supernatants were centrifuged more than once to avoid fatty layer contamination and their protein concentration was determined by the Lowry method. Two to ten micrograms of proteins from each sample was resolved by SDS-PAGE (10.0%) and immediately transferred to a nitrocellulose membrane using a transfer buffer (25 mM Tris, pH 8.8, 192 mM glycine with 20% (v/v) methanol). Nitrocellulose membranes were incubated in blocking solution (PBS with 0.05% (v/v) Tween 20 (PBST) containing 5% (w/v) skim milk or 1% (w/v) BSA (Sigma-Aldrich)) for 1 h at room temperature to block nonspecific binding. The blot was incubated overnight with anti-apoE antibody (Meridian, K23100R), anti-SR-B1 (abcam, ab52629), anti- lecithin cholesterina acyltransferase (LCAT) antibody (Aviva systems biology, ARP41695) or anti-β-actin (abcam, ab6276) as the primary antibodies, in blocking solution. The blot was washed 3 times for 10 min each in PBST at room temperature, incubated for 60 min in species appropriate horseradish peroxidase-conjugated secondary antibody (abcam, ab6728 for MsIgG and ab6721 for RbIgG) in blocking solution, and then washed 3 times in PBST, before being developed using the Super-Signal West Pico (Thermo Fisher Scientific Inc.).

### Quantitative RT-PCR

Total RNA was extracted from mouse liver and abdominal aorta (ab. aorta) using Trizol reagent (Invitrogen), and purified with an RNeasy Mini kit (Qiagen) according to the manufacturer’s instructions. cDNA was synthesized using a Quanti-Tect reverse transcription kit (Qiagen). Gene expression was measured by real-time RT-PCR using a LightCycler 480 (Roche Applied Science) and specific primers (Table S2). Averaged mRNA expression was normalized to GAPDH expression. Serial dilutions of a standard solution were included for each gene to generate a standard curve and allow calculation of the input amount of cDNA for each gene. A LightCycler 480 optical capillary tube was incubated at 95°C for 10 min to activate the FastStart Taq DNA polymerase. The run conditions were 55 cycles at 95°C for 10 sec, 59°C for 10 sec, and 72°C for 10 sec. Melting curves of each amplified gene were created to obtain PCR efficiency.

### Statistical Analysis

All data are presented as the mean±SEM. A two-tailed student *t* test was used to test for statistical significance. *P*<0.05 was considered statistically significant.

## Results

### Xanthohumol Prevents Cholesterol Accumulation in the Aortic Arch

Excess accumulation of cholesterol in an atherogenic region (*e.g.* aortic arch) is associated with atherosclerosis. The effect of oral administration of xanthohumol on total cholesterol accumulation in the aortic arch and liver was investigated. As shown in Figure 1A, T-Cho content in the aortic arch of control HCD-fed CETP-Tg mice was 2 fold higher than that in Chow diet fed CETP-Tg mice. However, oral administration of xanthohumol significantly reduced T-Cho content in the aortic arch induced by HDC to levels comparable with Chow diet-fed mice. Oral administration of xanthohumol to wild-type mice had no inhibitory effect on T-Cho accumulation in the aortic arch. These results suggested that xanthohumol suppressed the HCD-induced T-Cho accumulation by inhibiting CETP activity. Moreover, the endogenous T-Cho content in the liver of control HCD-fed CETP-Tg mice was 10 fold higher than that of normal diet-fed CETP-Tg mice. In addition, oral administration of xanthohumol significantly reduced HCD-induced T-Cho accumulation in liver (Figure 1B). In contrast to T-Cho accumulation in the aortic arch, endogenous T-Cho in the liver of wild-type mice was also significantly suppressed by oral administration of xanthohumol. These results suggested that the inhibitory effect of xanthohumol on T-Cho accumulation in liver was probably CETP-independent.

### Effect of Xanthohumol on Lipoprotein Profiles

As mentioned above, oral administration of xanthohumol significantly inhibited T-Cho accumulation in the aortic arch and liver of CETP-Tg mice, suggesting that CETP played an important role in the inhibitory effect of xanthohumol. Therefore, we investigated the effect of xanthohumol on serum CETP activity and HDL-C levels in CETP-Tg and wild-type mice. Serum T-Cho concentration in CETP-Tg and wild-type mice increased 2.5 and 2.9 fold, respectively, after intake of the HCD diet for 18 weeks. Oral administration of xanthohumol did not affect the serum T-Cho concentration when compared with the control group (Table S3), however, at 18 weeks, xanthohumol significantly increased serum HDL-C levels in CETP-Tg mice (*P*<0.05) and decreased it in wild-type mice (*P*<0.05) (Figure 2A and 2B). Similarly, xanthohumol significantly decreased the atherosclerosis index (AI, non-HDL-C/HDL-C) in CETP-Tg mice (*P*<0.001) (Table S3).

Xanthohumol also significantly inhibited serum CETP activity in CETP-Tg mice. We observed a negative correlation between CETP activity and HDL-C (%, HDL-C/T-Cho) (*R* = −0.38, *P*<0.05) (Figure 2C and 2D). These results suggested that xanthohumol increased HDL-C by inhibiting CETP activity. Furthermore, in CETP-Tg mice, CE content in the HDL fraction of the xanthohumol group was significantly higher than that of the control group. However, CE content in the serum of the xanthohumol group was significantly lower than that of the control group (Figure 2E and 2F), indicating that the increase in serum HDL-CE by xanthohumol might be due to the decreased transfer of CE from HDL to (V)LDL through its CETP inhibition. Xanthohumol decreased CE content in the HDL fraction of wild-type mice compared with the control group (*P*<0.05). Regulatory molecules other than CETP might also be involved in the change in CE transfer by xanthohumol.

### Effects of Xanthohumol on ApoE Levels in Serum and HDL Fraction

It has been reported that the serum apoE level was increased by inhibition of CETP activity [23], and that apoE enhanced cholesterol efflux [10]. Therefore, we investigated the effect of xanthohumol on apoE concentration in the serum and HDL-fraction. ApoE concentration in the serum and HDL-fraction was significantly increased by oral administration of xanthohumol to CETP-Tg mice. However, xanthohumol did not affect the apoE concentration in the serum and HDL-fraction of wild-type mice (Figure 3A). These results indicated that the increased serum apoE levels were mainly caused by an increase in the apoE concentration in the HDL fraction. ApoE gene expression in the liver of CETP-Tg mice was higher than that of wild-type mice, and this was not affected by xanthohumol (Figure 3B).

### Xanthohumol Up-regulated the Lipoprotein Receptor in Liver

As mentioned above, in wild-type mice, the HDL-CE concentration in serum was significantly decreased by oral administration of xanthohumol, however, xanthohumol significantly increased HDL-CE concentration in serum of CETP-Tg mice. To find out the cause of this discrepancy between CETP-Tg and wild-type mice, we investigated the effect of xanthohumol on LCAT and SR-B1 expression levels in liver. LCAT converts cholesterol to CE on the surface of lipoprotein, and HDL delivers cholesterol to the liver for disposal via SR-B1. Xanthohumol increased LCAT and SR-B1 protein expression levels in the liver of both CETP-Tg and wild-type mice (Figure 4A, 4B). These results suggested that the reduction in circulating HDL-CE in wild-type mice might be due to increased delivery of HDL-C to liver through the up-regulation of LCAT and SR-B1 by xanthohumol.

To further analyze the effects of xanthohumol on gene expressions associated with RCT, CETP, lipoprotein receptors and apolipoproteins were analyzed by quantitative RT-PCR. In CETP-Tg mice, liver gene expression of SR-B1 and LDL-R, which are both lipoprotein receptors, was significantly increased by xanthohumol (Figure 4C). In wild-type mice, liver gene expression of LDL-R and apoE was significantly increased by xanthohumol (Figure 4D). Xanthohumol did not show any effect on these gene expressions in the ab. aorta. These results indicated that xanthohumol up-regulates lipoprotein receptors in liver, but not in the ab. aorta.

## Discussion

Inhibition of CETP provides a useful strategy to raise HDL, the protective lipoprotein fraction in serum. We have already demonstrated that xanthohumol inhibits the CETP reaction *in vitro* [21]. Thus, in this study, we investigated the effect of xanthohumol on lipoprotein metabolism and anti-atherosclerosis induced by HCD using CETP-Tg mice. Before xanthohumol administration, the HDL-C/T-Cho ratio of CETP-Tg mice (50–60%) was much lower than that in wild-type mice (80%), suggesting that the high HDL-C/T-Cho ratio in wild-type mice was due to its CETP deficiency. The CETP-Tg mouse is a suitable experimental model to investigate the effect of certain drugs and foodstuffs on lipoprotein metabolism. We report for the first time that oral administration of xanthohumol ameliorates HCD-induced atherosclerosis by improving lipoprotein metabolism.

In CETP-Tg mice, HCD feeding significantly induced cholesterol accumulation in the aortic arch and liver. The increased cholesterol accumulation in aortic arch, induced by HCD intake, was suppressed by oral administration of xanthohumol in CETP-Tg mice but not in wild-type mice, suggesting that CETP inhibition by xanthohumol prevents cholesterol accumulation leading to atherosclerosis. Conversely, hepatic cholesterol accumulations were reduced by xanthohumol at 18 weeks in both strains. This should be caused from conserved mechanisms of xanthohumol in rodents other than CETP. In hepatic TG accumulation, there was no significant change in both strains (Figure S2), indicating that the reduction of hepatic T-Cho accumulation should not be resulted from non-specific inhibition of absorption in small intestine. We also have evaluated NPC1L1, intestinal cholesterol transporter, protein expression in Caco-2 cell line by Western blotting, but there was no significant difference between control and xanthohumol treatment (data not shown). Nozawa demonstrated that xanthohumol activated farnesoid × receptor (FXR) in the transient transfection assay and suggested that xanthohumol acts on FXR through a selective bile acid receptor modulator [24]. In wild-type mice FXR agonists increased fecal cholesterol excretion and PX20606, a type of FXR agonists, potently lowered total cholesterol and HDL-C even in CETP-Tg/LDL-R^−/−^ mice [25]. In our other experiment, Syrian hamsters were fed a high cholesterol (0.1%) and high fat diet (20%) (Table S4) for 2 weeks. Xanthohumol significantly increased the excretions of bile acid and T-Cho in feces when compared with the control group (*P*<0.001) (Figure S3). Thus the hepatic cholesterol decrease by xanthohumol in both strains, CETP-Tg mice and wild-type mice, might be attributed to cholesterol excretion via bile acid. It will be interesting to elucidate the effects of xanthohumol on FXR and ATP-binding cassette transporters 5/8 related to cholesterol excretion.

Xanthohumol also significantly inhibited serum CETP activity by 21% and increased serum HDL-C by 25% in CETP-Tg mice. Serum HDL-C concentration, measured after precipitation of (V)LDL by means of tungstophosphoric acid and magnesium chloride, was not changed by xanthohumol (data not shown). These results suggested that the increase in serum HDL-C concentration by xanthohumol, which was determined using 13% PEG, might be caused by the increase in apoE-rich HDL [9]. In this study, apoE protein levels in the serum and HDL-fraction were significantly increased by xanthohumol in CETP-Tg mice but not in wild-type mice. A recent study reported that anacetrapib promoted cholesterol efflux from macrophages to HDL and the increased HDL-C took the form of large HDL particles [26]. ApoE plays an important role in HDL particle expansion [27]. Therefore, apoE-rich HDL, caused by xanthohumol, may be due to enhancement of cholesterol efflux [10]. Oral administration of xanthohumol did not show any effect on CETP gene transcript in the liver and ab. aorta in CETP-Tg mice. We speculated that xanthohumol might directly inhibit the cholesterol transfer reaction by binding to the CETP protein itself or the CETP-lipid complex [21].

Many studies have recently reported that enhancement of RCT would have anti-atherosclerotic properties. Protein expression analyses of liver revealed that xanthohumol increased SR-B1 and LCAT expression in both mice strains. These results suggested that in wild-type mice, selective uptake of HDL-CE in the xanthohumol group might be due to up-regulation of SR-B1 expression by administration of xanthohumol. However, in CETP-Tg mice, the increase of hepatic SR-BI protein expression was a little weak (1.3 folds vs control group) (Figure 4A). To further analyze, we evaluated the SR-B1 expression level in CETP-Tg mice liver after 4 weeks HCD feeding. Relative mRNA expression of SR-B1 was not significant changed (control: 1.00±0.14, xanthohumol: 0.82±0.06, Mean±SE, n = 4 and 3, respectively), and SR-B1 protein expression was the same (control: 1.00±0.10, xanthohumol: 0.76±0.07, Mean±SE, n = 4 and 3, respectively). Zhang et al. also reported that FXR has previously been shown to induce SR-BI expression [28]. Also it remains possibility that SR-B1 expression was up-regulated by hepatic cholesterol. Thus, it seems a little difficult that SR-B1 increased by xanthohumol affects HDL turnover in CETP-Tg mice. Regarding LCAT, Yvan-Charvet et al. demonstrated that HDL-2 from subjects treated anacetrapib, CETP inhibitory drug, exhibited significant increases in apoE and LCAT proteins, and HDL-CE compared with control HDL-2, and indicated that a role of apoE acting in conjunction with LCAT is facilitating expansion of the CE core of HDL and permitting ongoing cholesterol efflux [29]. Although in CETP-Tg mice the increase of LCAT expression by xanthohumol found in liver, it might direct to cholesterol efflux as well as anacetrapib.

Quantitative RT-PCR analyses revealed that LDL-R significantly increased by xanthohumol in both strains, suggesting that the effect is conserved mechanism of xanthohumol in rodents independent of their lipoprotein profile. The increases of LDL-R expression should be attributed to the lowering in hepatic cholesterol to clear serum cholesterol. Also, in CETP-Tg mice, xanthohumol increased SR-B1 transcripts levels and it would be considered as compensation for uptake of HDL-C increased by xanthohumol via CETP inhibition. There remain unsolved about the discrepancy of the effect of xanthohumol on apoE expression between liver and ab. aorta in wild-type mice.

CETP inhibitors are expected to be promising anti-atherosclerotic agents through their enhancement of serum HDL-C concentration. However, there is concern that the increase in HDL-C by CETP inhibition might lead to an impediment in cholesterol reverse transport. The efficacy of a CETP inhibitor as an anti-atherosclerotic agent is now a controversial issue. In this study we demonstrated that xanthohumol reduces cholesterol accumulation in atherogenic region via CETP inhibition. For this reason, we expect that xanthohumol might be a promising agent to prevent and ameliorate the development of atherosclerosis.

## Supporting Information

Figure S1 **Effect of 13% polyethylene glycol on CETP-transgenic mice.** Electrophoresis of serum (Left) and HDL-fraction (Right) lipoproteins from 9-week old mice. Gels were visualized by total cholesterol enzymatic staining (HELENA, Japan).
(TIF)

Figure S2 **Hepatic TG accumulation at 18 weeks.** Data are presented as TG amount in liver. (N = 15; CETP-Tg mice control, N = 18; CETP-Tg mice xanthohumol, N = 12; CETP-Tg mice Chow, N = 10; wild-type mice control, N = 7; wild-type mice xanthohumol) Means±SEM.
(TIF)

Figure S3 **Bile acid secretion in Syrian hamster fed high cholesterol and high fat diet over 2 weeks.** Xanthohumol was administered at 1 mg/kg⋅BW dose by osmotic pumps (alzet, Cupertino, CA) subcutaneously. Feces were collected for 7 days before anatomy and weighed. For bile acid and fecal T-Cho analysis, lyophilized 50 mg feces were extracted as previously described [1], [2] and measured by commercial kit (Test-Wako). (N = 8; control, N = 8; xanthohumol, N = 8; Chow) Means±SEM.
(TIF)

Table S1 **Components of the high cholesterol diet (HCD).**
(TIF)

Table S2 **List of primers used in this study.** ApoA-Forward, apoA-Reverse, apoE-Forward and apoE-Reverse were purchased from TaKaRa Bio Inc. (Shiga, Japan).
(TIF)

Table S3 **Serum lipid and CETP activity.** Abbreviations are as follows: nonHDL-C, non HDL-cholesterol; AI, atherosclerosis index; FC: free cholesterol. nonHDL-C was calculated by subtracting HDL-C from T-Cho. AI was calculated as nonHDL-C/HDL-C. HDL-CE was calculated by subtracting HDL-FC from HDL-C. HDL-C, HDL-TG and HDL-PL were analyzed using test-wako kits on the HDL-fraction treated with 13% PEG solution. (N = 15; CETP-Tg mice control, N = 16; CETP-Tg mice xanthohumol, N = 10; CETP-Tg mice Chow, N = 3; wild-type mice control, N = 8; wild-type mice xanthohumol) Means±SEM. *P<0.05, **P<0.01, ***P<0.001.
(TIF)

Table S4 **Components of the high cholesterol and high fat diet.**
(TIF)

## References

[1] Gordon T, Castelli WP, Hjortland MC, Kannel WB, Dawber TR (1977) High density lipoprotein as a protective factor against coronary heart disease. The Framingham Study. Am J Med. 62: 707–714. 10.1016/0002-9343(77)90874-9 193398

[2] Libby P, Aikawa M (2002) Stabilization of atherosclerotic plaques: new mechanisms and clinical targets. Nat Med. 8: 1257–62. 10.1038/nm1102-1257 12411953

[3] Barter P, Gotto AM, LaRosa JC, Maroni J, Szarek M, et al (2007) Treating to New Targets Investigators. HDL-C, very low levels of LDL cholesterol, and cardiovascular events. N Engl J Med. 357: 1301–10. 10.1056/NEJMoa064278 17898099

[4] Spieker LE, Ruschitzka F, Lüscher TF, Noll G (2004) HDL and inflammation in atherosclerosis. Curr Drug Targets Immune Endocr Metabol Disord. 4: 51–7. 10.2174/1568008043340044 15032626

[5] Lewis GF, Rader DJ (2005) New insights into the regulation of HDL metabolism and reverse cholesterol transport. Circ Res. 96: 1221–32. 10.1161/01.RES.0000170946.56981.5c 15976321

[6] Tall AR (1993) Plasma cholesteryl ester transfer protein. J Lipid Res. 34: 1255–74. 8409761

[7] Brown ML, Inazu A, Hesler CB, Agellon LB, Mann C, et al (1989) Molecular basis of lipid transfer protein deficiency in a family with increased high-density lipoproteins. Nature. 342: 448–451. 10.1038/342448a0 2586614

[8] Inazu A, Brown ML, Hesler CB, Agellon LB, Koizumi J, et al (1990) Increased high-density lipoprotein levels caused by a common cholesteryl-ester transfer protein gene mutation. N Engl J Med. 323: 1234–8. 10.1056/NEJM199011013231803 2215607

[9] Chiba H, Akita H, Tsuchihashi K, Hui SP, Takahashi Y, et al (1997) Quantitative and compositional changes in high density lipoprotein subclasses in patients with various genotypes of cholesteryl ester transfer protein deficiency. J Lipid Res. 38: 1204–16. 9215548

[10] Matsuura F, Wang N, Chen W, Jiang XC, Tall AR (2006) HDL from CETP-deficient subjects shows enhanced ability to promote cholesterol efflux from macrophages in an apoE- and ABCG1-dependent pathway. J Clin Invest. 116: 1435–42. 10.1172/JCI27602 PMC1451209 16670775

[11] Okamoto H, Yonemori F, Wakitani K, Minowa T, Maeda K, et al (2000) A cholesteryl ester transfer protein inhibitor attenuates atherosclerosis in rabbits. Nature 406: 203–207. 10910363 10.1038/35018119

[12] Clark RW, Sutfin TA, Ruggeri RB, Willauer AT, Sugarman ED, et al (2004) Raising high-density lipoprotein in humans through inhibition of cholesteryl ester transfer protein: an initial multidose study of torcetrapib. Arterioscler Thromb Vasc Biol. 24: 490–7. 10.1161/01.ATV.0000118278.21719.17 14739125

[13] Brown ML, Inazu A, Hesler CB, Agellon LB, Mann C, et al (1989) Molecular basis of lipid transfer protein deficiency in a family with increased high-density lipoproteins. Nature. 342: 448–451. 10.1038/342448a0 2586614

[14] Quinet E, Tall A, Ramakrishnan R, Rudel L (1991) Plasma lipid transfer protein as a determinant of the atherogenicity of monkey plasma lipoproteins. J Clin Invest. 87: 1559–66. 10.1172/JCI115169 PMC295238 2022728

[15] Marotti KR, Castle CK, Boyle TP, Lin AH, Murray RW, et al (1993) Melchior GW. Severe atherosclerosis in transgenic mice expressing simian cholesteryl ester transfer protein. Nature. 364: 73–5. 10.1038/364073a0 8316302

[16] Brown MS, Goldstein JL (1976) Receptor-mediated control of cholesterol metabolism. Science 191: 150–4. 174194 10.1126/science.174194

[17] Nicholls SJ (2011) Apo A-I Modulating Therapies. Curr Cardiol Rep. 13: 537–43. 10.1007/s11886-011-0223-0 21947768

[18] Dorn C, Kraus B, Motyl M, Weiss TS, Gehrig M, et al (2010) Schölmerich J, Heilmann J, Hellerbrand C. Xanthohumol, a chalcon derived from hops, inhibits hepatic inflammation and fibrosis. Mol Nutr Food Res. 54: S205–13. 10.1002/mnfr.200900314 20087858

[19] Tabata N, Ito M, Tomoda H, Omura S (1997) Xanthohumols, diacylglycerol acyltransferase inhibitors, from Humulus lupulus. Phytochemistry. 46: 683–7. 10.1016/s0031-9422(97)00157-x 9366096

[20] Strathmann J, Klimo K, Sauer SW, Okun JG, Prehn JH, et al (2010) Gerhäuser C. Xanthohumol-induced transient superoxide anion radical formation triggers cancer cells into apoptosis via a mitochondria-mediated mechanism. FASEB J. 24: 2938–50. 10.1096/fj.10-155846 20335224

[21] Hirata H, Takazumi1 K, Segawa1 S, Okada1 Y, Kobayashi1 N, et al (2012) Xanthohumol, a prenylated chalcone from Humulus lupulus L., inhibits cholesteryl ester transfer protein. Food Chem. 134: 1432–37. 10.1016/j.foodchem.2012.03.043 25005963

[22] Folch J, Lees M, Sloane Stanley GH (1957) A simple method for the isolation and purification of total lipids from animal tissues. J Biol Chem. 226: 497–509. 13428781

[23] Zhang B, Fan P, Shimoji E, Xu H, Takeuchi K, et al (2004) Inhibition of cholesteryl ester transfer protein activity by JTT-705 increases apolipoprotein E-containing high-density lipoprotein and favorably affects the function and enzyme composition of high-density lipoprotein in rabbits. Arterioscler Thromb Vasc Biol. 24: 1910–5. 10.1161/01.ATV.0000143389.00252.bc 15331428

[24] Hajime N (2005) Xanthohumol, the chalcone from beer hops (Humulus lupulus L.), is the ligand for farnesoid × receptor and ameliorates lipid and glucose metabolism in KK-A(y) mice. Biochem Biophys Res Commun. 336: 754–61. 10.1016/j.bbrc.2005.08.159 16140264

[25] Hambruch E, Miyazaki-Anzai S, Hahn U, Matysik S, Boettcher A, et al.. (2012) Synthetic FXR Agonists Induce HDL-Mediated Transhepatic Cholesterol Efflux in Mice and Monkeys and Prevent Atherosclerosis in CETP Transgenic LDLR−/− Mice. J Pharmacol Exp Ther. [Epub ahead of print]. 10.1124/jpet.112.196519 PMC11047796 22918042

[26] Schwartz GG (2012) New Horizons for Cholesterol Ester Transfer Protein Inhibitors. Curr Atheroscler Rep. 14: 41–8. 10.1007/s11883-011-0217-9 22083134

[27] Mahley RW, Huang Y, Weisgraber KH (2006) Putting cholesterol in its place: apoE and reverse cholesterol transport. J Clin Invest. 116: 1226–9. 10.1172/JCI28632 PMC1451226 16670767

[28] Zhang Y, Yin L, Anderson J, Ma H, Gonzalez FJ, et al (2010) Identification of Novel Pathways That Control Farnesoid × Receptor-mediated Hypocholesterolemia. J Biol Chem. 285: 3035–43. 10.1074/jbc.M109.083899 PMC2823426 19996107

[29] Yvan-Charvet L, Kling J, Pagler T, Li H, Hubbard B, et al (2010) Cholesterol efflux potential and antiinflammatory properties of high-density lipoprotein after treatment with niacin or anacetrapib. Arterioscler Thromb Vasc Biol. 30: 1430–8. 10.1161/ATVBAHA.110.207142 PMC2917780 20448206

