# Supplementary figures and images for: Xanthohumol Prevents Atherosclerosis by Reducing Arterial Cholesterol Content via CETP and Apolipoprotein E in CETP-Transgenic Mice
Source: PLoS One. 2012 Nov 16;7(11):e49415. doi: 10.1371/journal.pone.0049415 (PMC3500296; doi:10.1371/journal.pone.0049415)

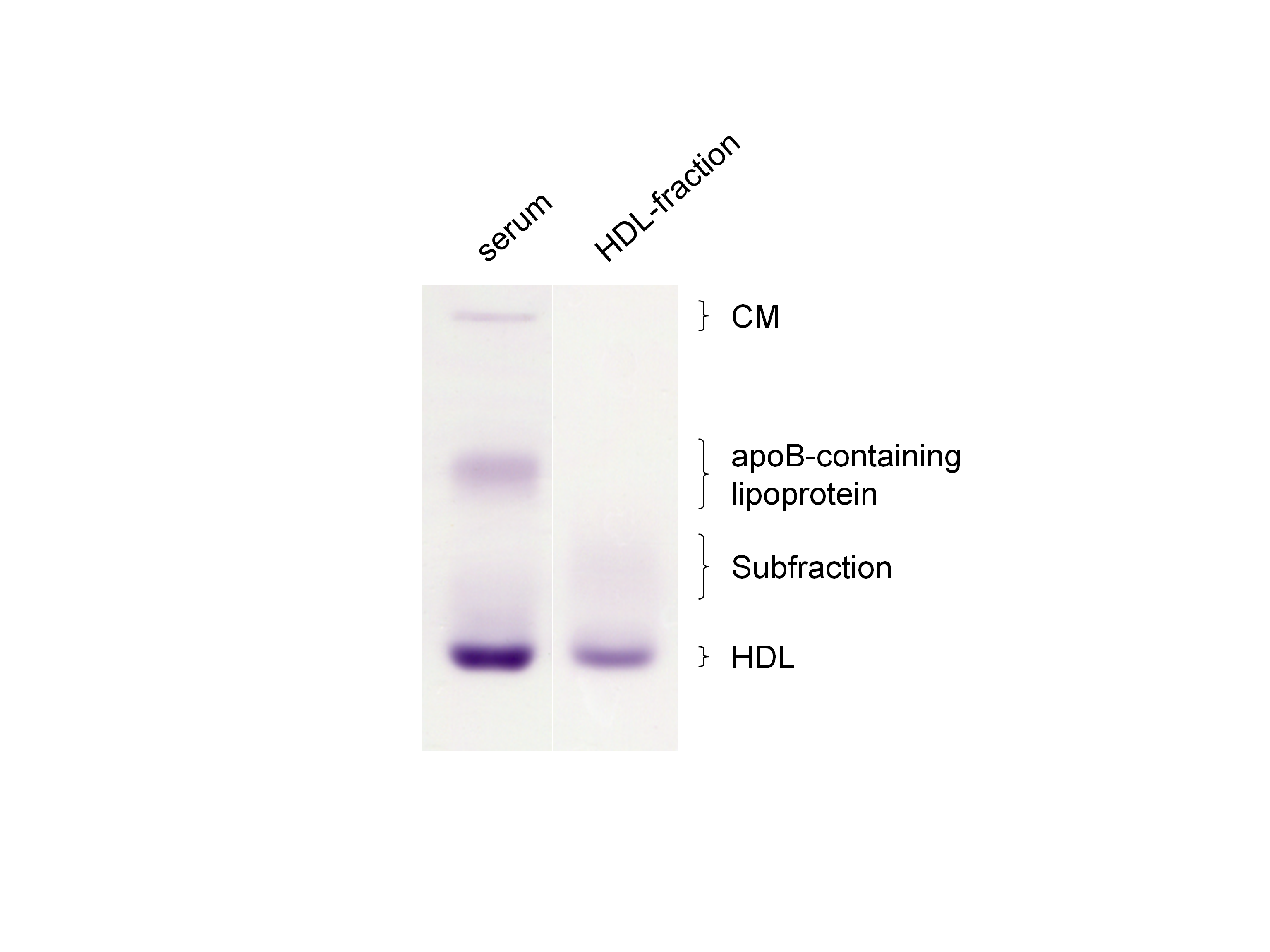

Supplement: Figure S1 — Effect of 13% polyethylene glycol on CETP-transgenic mice. Electrophoresis of serum (Left) and HDL-fraction (Right) lipoproteins from 9-week old mice. Gels were visualized by total cholesterol enzymatic staining (HELENA, Japan). (TIF) [file pone.0049415.s001.tif]
